# Understanding the function and dysfunction of the immune system in lung cancer: the role of immune checkpoints

**DOI:** 10.7497/j.issn.2095-3941.2015.0029

**Published:** 2015-06

**Authors:** Niki Karachaliou, Maria Gonzalez Cao, Cristina Teixidó, Santiago Viteri, Daniela Morales-Espinosa, Mariacarmela Santarpia, Rafael Rosell

**Affiliations:** ^1^Instituto Oncológico Dr Rosell, Quiron Dexeus University Hospital, Barcelona 08028, Spain; ^2^Pangaea Biotech, Barcelona 08028, Spain; ^3^Medical Oncology Unit, Human Pathology Department, University of Messina, Messina 98122, Italy; ^4^Catalan Institute of Oncology, Hospital Germans Trias i Pujol, Badalona 08916, Spain; ^5^Molecular Oncology Research (MORe) Foundation, Barcelona 08028, Spain; ^6^Germans Trias i Pujol Health Sciences Institute and Hospital, Campus Can Ruti 08916, Spain

**Keywords:** Lung cancer, immunotherapy, immune checkpoint, program death-ligand 1 (PD-L1), program death-1 (PD-1)

## Abstract

Survival rates for metastatic lung cancer, including non-small cell lung cancer (NSCLC) and small cell lung cancer (SCLC), are poor with 5-year survivals of less than 5%. The immune system has an intricate and complex relationship with tumorigenesis; a groundswell of research on the immune system is leading to greater understanding of how cancer progresses and presenting new ways to halt disease progress. Due to the extraordinary power of the immune system—with its capacity for memory, exquisite specificity and central and universal role in human biology—immunotherapy has the potential to achieve complete, long-lasting remissions and cures, with few side effects for any cancer patient, regardless of cancer type. As a result, a range of cancer therapies are under development that work by turning our own immune cells against tumors. However deeper understanding of the complexity of immunomodulation by tumors is key to the development of effective immunotherapies, especially in lung cancer.

## Introduction

Lung cancer is one of the leading causes of cancer-related death globally. Non-small cell lung cancer (NSCLC) is the most common type, accounting for nearly 85% of all newly diagnosed cases^1^. Most patients with NSCLC either present with metastatic disease or experience disease recurrence despite undergoing treatment for seemingly localized disease, underscoring the systemic nature of this disease. Cytotoxic chemotherapy regimens developed over the past few decades have produced only modest improvements in survival in metastatic NSCLC. A small subset of patients with tumors driven by activating mutations in the gene encoding epidermal growth factor receptor (EGFR) or rearrangements in the gene coding for anaplastic lymphoma kinase (ALK) benefit substantially from specific targeted therapies^2^^-^^4^. However, most of these patients eventually succumb to tumor progression within a few years of diagnosis. Thus therapies that obtain long lasting disease control are urgently needed.

The immune system plays an important role in controlling and eradicating cancer. Nevertheless, in the setting of malignancy, multiple mechanisms of immune suppression may exist that prevent effective antitumor immunity. Antibody therapy directed against several negative immunologic regulators is currently demonstrating significant success and is likely to become a major component of treatment for patients with a variety of malignancies. Therefore, this review focuses on the role of immune system in cancer and indeed lung cancer.

## What is an immune checkpoint?

Thymus-derived lymphocytes (T-lymphocytes, T-cells) activation and expansion are necessary for an effective acquired immune response. Spontaneous lymphocytic infiltrates can be consistently observed in a variety of tumors. CD4 T-cells and CD8 T-cells make up the majority of T-lymphocytes. Interferon-γ producing CD8 T cells play an important role in inhibiting and killing tumor cells and impeding tumor growth. Interleukin-12 and granulocyte-macrophage colony-stimulating factor (GM-CSF) induce the activation of tumor-resident CD8 T effector/memory cells (Tem) followed by cytotoxic CD8 T effector cell expansion, a population that is a potent *in situ* resource for successful reactivation of systemic antitumor T cell immunity^5^. Amongst the many factors CD8 T cells produced, interferon-γ seems to be one of most significant cytokines in preventing and suppressing the development of cancers. In addition, the cytotoxic effects of CD8 T cells may also directly mediate death of tumor cells^6^.

After being activated and differentiated into distinct effector subtypes, CD4 T-cells play a major role in mediating immune response through the secretion of specific cytokines. These cells have multiple functions, ranging from activation of the cells of the innate immune system, B-lymphocytes, cytotoxic T-cells, as well as non-immune cells, and also play a critical role in suppression of immune reaction. Ongoing studies have identified new subsets of CD4 cells besides the classical T-helper 1 and 2 cells, like T-helper 17, follicular helper T-cell, induced T-regulatory cells (Treg), and the regulatory type 1 cells as well as the potentially distinct T-helper 9^7^. Tregs, originally termed suppressive T-cells, were first described in the early 1970s as thymus-derived lymphocytes that tolerized bone marrow-derived lymphocytes to antigenic challenge^8^^,^^9^. Subsequent research demonstrated that T-cells expressing CD4 and CD25 [the alpha chain of interleukin-2 (IL-2) receptor] from tumor-bearing mice abrogated tumor rejection^10^^-^^14^. It was 10 years later that Sakaguchi and colleagues ascertained that CD25 could be used to identify these suppressive cells^15^. Later studies from the same laboratory established the forkhead box P3 (FoxP3) transcription factor as both a key intracellular marker of CD4^+^ CD25^+^ Tregs and a necessary factor for development and proper function of these cells^16^.

**Figure 1:**
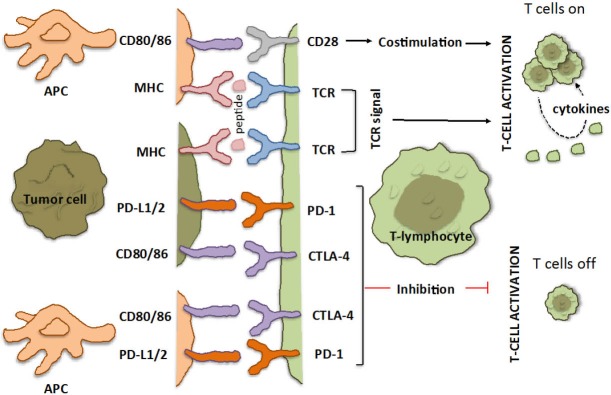
T-cell interaction with APC and tumor cells: the immune checkpoints CTLA-4 and PD-1/PD-L1. Depicted are various ligand-receptor interactions between T-cells, APCs and cancer cells that regulate the T-cell response to antigen. Activation of T-cells is a two-step process that requires recognition of specific peptides presented by MHC on the surface of cancer cells through their TCR, as well as a co-regulatory signal delivered by the CD28 family of receptors (the so-called immune checkpoints). The co-regulatory signal promotes T-cell clonal expansion, cytokine secretion, and functional activity of the T-cell. In the absence of this signal (even in the presence of a target peptide), T-cells fail to respond effectively and are functionally inactivated. This is designed as a fail-safe mechanism to ensure that the immune system is activated at the appropriate time in order to limit collateral damage to normal tissue and minimize the possibility of chronic autoimmune inflammation. Checkpoint pathways regulate these coregulatory signals and can be either stimulatory (switching T-cells on) or inhibitory (switching them off). CTLA-4 and PD-1 deliver inhibitor signals. CTLA-4 negatively regulates T-cell activation by binding to B7 molecules (CD80/86) on the surface of APC or tumor cell. Conversely, when these B7 molecules bind to CD28 they generate the opposite effect, activating signals. When PD-1 binds to either of its ligands (PD-L1 or PD-L2), which are primarily expressed within inflamed tissues and the tumor microenvironment, it results in inhibition of T-cell activity. APC, antigen-presenting cell (dendritic cell, macrophage or any cell that expresses antigen); TCR, T-cell receptor; MHC, major histocompatibility complex.

One of the key attributes is how the T-cells activate and distinguish “self” from “non-self” molecules. A series of positive and negative costimulatory receptors are expressed on a T-cell at variable levels according to the timing and circumstances of the immune response. The efficiency with which CD4 T-cells direct an immune response demands that proper regulatory measures are in place to prevent immune hyperactivation leading to autoimmune disease. This is very important especially for organs like the lungs that have large mucosal and gas-exchanging surfaces which are constantly exposed to the environment^17^. Such a critical process involves presentation of antigens to T-cells by antigen presenting cells (APC) and is highly regulated by molecules on T-cells and APC as well as tumor and stromal cells, known as immune checkpoints. Recognition of antigen- major histocompatibility complex (MHC) complexes by the T-cell antigen receptor is not sufficient for activation of naïve T-cells. Additional costimulatory signals are required and are provided by the engagement of CD28 on the T-cell surface with B7 molecules (CD80 and CD86) on the APC^18^^,^^19^ (**Figure 1**). The role of immune checkpoints is not only to trigger a sufficient immune response but also to inhibit stimulation to ensure the inductive immune response is not excessive. In fact, these immune checkpoints, usually referred to as molecules of inhibitory pathways in the immune system, are crucial for maintaining self-tolerance and modulating physiological immune responses in the periphery, in order to avoid or minimize tissue damage from excess reactions.

The CD28 family of cell surface receptors [CD28, cytotoxic T-lymphocyte-associated antigen 4 (CTLA-4), inducible costimulator (ICOS), program death-1 (PD-1), and B- and T-lymphocyte attenuator (BTLA)] plays a critical role in controlling the adaptive arm of the immune response and controlling T-cell activation. The counterpart (ligand) for CD28 is the “B7 family”, containing B7-1 (CD80) and B7-2 (CD86), which are usually present on APC. Although there is structural similarity between members of the CD28 family, functional heterogeneity is observed. For instance, ligation of CD28 and ICOS promotes T-cell activation, whereas engagement of CTLA-4, PD-1, and BTLA inhibits T-cell activation^20^. Other regulators of T-cell activation have recently been characterized and may have important roles. These include T-cell immunoglobulin and mucin domain-containing protein 3 (TIM3; also known as HAVCR2), lymphocyte activation gene-3 (LAG-3) and V-domain immunoglobulin suppressor of T-cell activation (VISTA)^21^^-^^23^.

CTLA-4 is expressed exclusively on T-cells and shares identical ligands (CD80 and CD86) with the T-cell co-stimulatory receptor CD28. When the T-cell receptor (TCR) is engaged by cognate antigen, CD28 induces T-cell activation. CTLA-4 has a much higher overall affinity for both ligands and inhibits the activation of T-cells by outcompeting CD28 in binding CD80 and CD86. At the same time, CTLA-4 activates the Src homology region 2 domain-containing phosphatase-2 (SHP2) and protein phosphatase 2A (PP2A) and counteracts kinase signals induced by TCR and CD28, sequestrates CD80 and CD86 from CD28 engagement, and actively removes CD80 and CD86 from the APC surface.

PD-1 signaling involves binding to several discrete ligands, including PD-L1 (also known as B7-H1 and CD274) and PD-L2 (also known as B7-DC and CD273), as well as to the co-stimulatory molecule CD80. The PD-1/PD-L1 interaction inhibits T-lymphocyte proliferation, survival and effector functions (cytotoxicity, cytokine release), induces apoptosis of tumor-specific T-cell and promotes differentiation of CD4 T-cells into Tregs and tumor cell resistance to cytotoxic T-lymphocytes (CTL) attack^21^. Because many tumors are highly infiltrated with Tregs that probably further suppress effector immune responses, blockade of the PD-1 pathway may also enhance antitumor immune responses by diminishing the number and/or suppressive activity of intratumoral Tregs. Chemnitz *et al*.^24^ revealed that the ability of PD-1 to block T-cell activation correlates with recruitment of SHP-1 and SHP-2. Indeed, PD-1 has a cytoplasmic immunoreceptor tyrosine based inhibitory motif (ITIM), as well as an immunoreceptor tyrosine-based switch motif (ITSM), and has been found to be capable of recruiting the phosphatases SHP-1 and SHP-2. Recruitment of SHP-1 and SHP-2 to ITIM within the PD-1 cytoplasmic tail inhibits positive signaling events downstream of the TCR, mainly PI3K/AKT activation^25^.

SHP-1 and SHP-2 are highly related tyrosine phosphatases that serve very distinct roles in signal transduction. SHP-1 expression is largely confined to hemopoietic cells and is thought to act as a negative regulator of STAT3 and other signaling pathways. SHP1 is encoded by the PTPN6 gene and the regulatory factor X-1 (RFX-1) is one transcription factor that can activate SHP-1 transcription^26^. SHP-2, in contrast, is widely expressed and generally acts in a positive manner to transduce signals from receptor protein tyrosine kinases. For instance, an established role of SHP-2 in EGFR or ALK signaling is to mediate ERK1/2 activation. However, SHP-2 also has been shown to inhibit the JAK-STAT signaling pathway^27^^-^^29^.

## Immune response and cancer

Immunotherapies that boost the ability of endogenous T-cells to destroy cancer cells have demonstrated therapeutic efficacy in a variety of human malignancies. In 2010, the field was revitalized by a landmark randomized clinical trial that demonstrated that treatment with ipilimumab, an antibody targeting CTLA-4, improved overall survival (OS) of patients with metastatic melanoma^30^. Recent studies have demonstrated that T-cell–based immunotherapies are also effective in a range of other human malignancies. In particular, clinical trials of antibodies that interfere with PD-1 have shown clinical activity in tumor types as diverse as lung, bladder, stomach, renal cell, and head and neck cancer, as well as melanoma and Hodgkin’s lymphoma^31^.

T-cells in tumors—the so-called tumor infiltrating lymphocytes (TIL) have been studied intensively over the past years. The first evidence that T-cells could kill tumor cells was provided by L.R. Freedman and colleagues in 1972^32^. Numerous studies suggest a positive prognostic impact of TIL but this still needs to be verified in large multi-center studies^33^. At present there is very limited knowledge as to why some tumors are heavily infiltrated by T-cells whereas others are not. Studies from the laboratory of Robert Schreiber have suggested the “Three Es of cancer immunoediting”^34^, or three phases of interaction between tumor and immune system: immune-Elimination of cancer cells, immune Equilibrium between cancer cells and cells of the immune system and immune Escape by cancer cells^34^. However, this notion is still unclear and TILs display a wide range of different phenotypes. Studies have shown that CD8 T-cells at the tumor site display markers of T-cell exhaustion to a higher extent than T-cells in the blood or from normal adjacent tissue^35^^,^^36^. In melanomas, CD8 and CD4 TILs display high expression of PD-1 and CTLA-4. Furthermore, the PD-1 positive fraction of the TILs displays impaired effector functions^35^.

## Tumor and PD-L1 expression

Tumor cells can activate PD-L1 expression via multiple oncogenic signaling pathways involving IFN-γ/JAK2/IFN^37^, PI3K^38^, ALK/STAT3^39^, MEK/ERK/STAT1, MYD88/TRAF6^40^ or exposure to inflammatory cytokines such as IFN-γ^41^ produced by infiltrating immune cells. In breast cancer, PD-L1 expression is strongly associated with proliferative Ki-67 expression and cell cycle progression independent of host PD-1^42^. In human glioma, loss of the tumor suppressor gene phosphatase and tensin homolog (PTEN) has been correlated with enhanced PD-L1 expression^38^. Similarly, in colorectal cancer, miR-20b, -21 and 130 inhibited PTEN expression, resulting in PD-L1 overexpression^43^. T-cell lymphoma cells carrying the oncogenic nucleophosmin (NPM)-ALK, involved in malignant transformation, induce high levels of PD-L1 expression via STAT3 and ERK activation^39^^,^^44^.

Abnormal expression of PD-L1 has been described in 19%-100% of NSCLCs and is associated with poor prognosis^45^^-^^48^. Reliable biomarkers associated with response to PD-1 blockade remain poorly understood^49^. Simultaneous activation of KRAS and inactivation of serine-threonine kinase 11 (also known as LKB1) induce lung squamous cell carcinoma formation^50^. Activation of the EGFR pathway might be involved in suppressing the immune response in murine melanoma models either through activating Tregs cells or reducing the levels of the T-cell chemoattractant^49^. Interestingly, Akbay *et al*.^51^ found that activation of the EGFR pathway induced PD-L1 expression to help NSCLC tumors to remodel tumor microenvironment to trigger immune escape and link tumor response to PD-1 inhibition. This role of EGFR signaling was independent of its effects on cell proliferation and survival, suggesting that the combination of PD-1 blockade with EGFR TKIs may be a promising therapeutic strategy to extend the duration of treatment response and delay development of resistance to EGFR inhibitors^51^. D’Incecco *et al*.^52^ found that PD-L1 positive NSCLC patients had higher sensitivity to EGFR-TKIs, longer time to progression and OS than PD-1 negative patients. They also reported that PD-L1 positive status was significantly associated with presence of EGFR mutations^52^. In the study of Azuma *et al*.^53^, inhibition of EGFR signaling by erlotinib down-regulated surface expression of PD-L1 in EGFR mutation-positive NSCLC cells, but not in the EGFR wild-type cells. In contrast, Mu *et al*.^47^ found no significant correlation between PD-L1 expression and EGFR/KRAS/BRAF/ALK expression in stage I NSCLC patients, similar to Zhang *et al*.^54^, who found no significant relationship between PD-L1 expression and EGFR/KRAS expression in lung adenocarcinoma. At the 2015 ASCO Annual Meeting, median progression free survival (PFS) and OS for EGFR TKIs were similar between PD-L1 positive and PD-L1 negative patients at baseline. Also, median PFS for ALK TKIs was similar in PD-L1 positive and PD-L1 negative patients at baseline, but median OS was shorter among PD-L1 positive patients. Expression was dynamic, with changes in PD-L1 expression and immune infiltrates observed over time and/or following treatment^55^.

## Cancer immunotherapy in clinical practice

Three new immune checkpoint agents have now been approved by the U.S. Food and Drug Administration (FDA) for the treatment of melanoma^31^. The list of cancers that can be targeted with immunotherapy is growing and there are high expectations that immune checkpoint agents will also be approved for treatment of patients with lung, kidney, bladder and prostate cancer, as well as lymphoma and many other tumor types. Immune checkpoints inhibitors target molecules that regulate T cells rather than the T cells themselves in order to reverse the activation of inhibitory pathways and release antitumor T-cell responses.

Two phase III clinical trials with anti-CTLA-4 (ipilimumab) were conducted in patients with advanced melanoma and demonstrated improved OS with the drug^30^^,^^56^. Anti-CTLA-4, having more mature survival data than other agents, leads to durable clinical responses that can last a decade and more, but only in a fraction of patients. A recent analysis indicated survival of 10 years or more for a subset of patients^57^. Ipilimumab was approved in 2011.

Pembrolizumab and nivolumab, two antibodies against PD-1 were approved in September and December 2014, respectively, for treatment of metastatic melanoma^31^. A phase I clinical trial with pembrolizumab led to response rates of almost 38% in patients with advanced melanoma, and a subsequent study reported an overall response rate of 26% in patients who had progressive disease after prior ipilimumab treatment^58^^,^^59^. In a phase III trial, nivolumab improved OS of patients with metastatic melanoma in comparison with dacarbazine chemotharpy^59^. According to the results of the CheckMate 057 trial presented at the 2015 ASCO Annual Meeting, nivolumab is the first PD-1 inhibitor to significantly improve OS in comparison with docetaxel, in previously treated patients with advanced non-squamous NSCLC with 27% reduction in risk of death and significantly improved overall response rate. Tumor PD-L1 expression was found to be predictive of nivolumab benefit^60^. Nivolumab was FDA approved in March 2015 for patients with previously treated advanced or metastatic NSCLC based on a phase III clinical trial which reported an improvement in OS for patients treated with nivolumab as compared to patients treated with docetaxel chemotherapy^31^. In addition, nivolumab was recently found to be the first PD-1 inhibitor to demonstrate a survival benefit versus standard-of-care docetaxel in previously treated patients with advanced squamous NSCLC with 41% reduction in risk of death; benefit was independent of PD-1 expression^61^.

## Biomarkers and response to immunotherapy; neoantigen load as a potential biomarker for cancer immunotherapy

There are ongoing studies to identify predictive biomarkers to select patients for treatment with a particular agent, but this is complicated by the complexity of the immune response. The expression of PD-L1 in cancer cells is an obvious candidate as it can directly turn off the immune response by inhibiting the activity of cytotoxic T-cells infiltrating the tumor. However, PD-L1 expression in tumor cells has little predictive power. Tumeh *et al*.^62^ established a set of conditions that correlates with good response of patients with melanoma to pembrolizumab therapy. These include the presence of cytotoxic T-cells in the tumor, the expression of PD-L1 and PD-1 in immune cells in the tumor margin, and less complexity (in terms of antigen receptors) in the tumor T-cell population^62^. Herbst *et al*.^63^ also observed that PD-L1 expression in immune cells is a good biomarker of response to immunotherapy.

Blockade of CTLA-4 and PD-1 has resulted in durable responses in many patients^30^^,^^64^. However it remains unclear why some have only transient or no response. A major hurdle in tumor immunotherapy is the fact that mechanisms of self-tolerance that prevent autoimmunity also impair T-cell responses against tumors. The nature of the antigens that allow the immune system to distinguish cancer cells from non-cancer cells has long remained obscure. Every tumor contains hundreds or thousands of somatic mutations and certain types of tumors display many more or less mutations. Melanomas and lung cancers are the outliers and contain approximately 200 nonsynchronous mutations per tumor, associated with environmental exposure to ultraviolet light and smoking^65^. It seems that response to immune-based drugs may be written in tumor DNA. Tumors with a high somatic mutation load are more likely to respond to immunotherapy as, in theory, they would have a higher diversity of neoantigens that can trigger an immune response when the CTLA-4/PD-1 inhibition is bypassed. In NSCLC patients treated with anti–PD-1, mutational load shows a strong correlation with clinical response^66^. Likewise, in melanoma patients treated with ipilimumab, long-term benefit is also associated with a higher mutational load, although the effect appears less profound in this setting^67^. In the study of Snyder and colleagues^67^, mutational burden was higher in patients with a sustained clinical benefit than in those without. While the data indeed show that high mutation load correlates with responsiveness to therapy in many cases, surprisingly some tumors with a high load of somatic mutations fail to respond to checkpoint blockade. Therefore, quality not quantity of mutations has the strongest predictive value. A number of tetrapeptide sequences common to patients with sustained clinical benefit, but completely absent in patients with a minimal or no benefit, were homologous to viral and bacterial antigens^67^. An interesting interpretation of these data is that the neoantigen-specific T-cell response is preferentially directed toward a subset of mutant sequences, something that could facilitate bioinformatic identification of neoantigens for therapeutic targeting^68^. However, other studies have not found the profound bias toward these tetrapeptide signatures that would be predicted if their role was central to the tumor-specific T-cell response, meaning that the identified tetrapeptide motifs may play a different role^69^.

## Conclusion

Cancer immunotherapy relies on the ability of the immune system to identify and destroy tumor cells and elicit a long-lasting memory of this interaction. Various strategies are being developed to enhance anti-tumor immune responses, with a recent focus on antagonists of inhibitory signaling pathways to overcome immune checkpoints. Existing therapies are also being investigated for their ability to induce an anti-tumor immune response, something which could lead to administration of combination therapies providing a more efficacious and durable response. However, there are issues that remain to be understood. Soon many cancer immunotherapies will be made available, many combinations will be possible, and this choice will be quite challenging from a clinical, regulatory, and reimbursement perspective. Biomarkers and companion diagnostics may also play a big role in guiding the way, as will a deepening understanding of immunotherapy mechanisms and cancer response.
